# Eosinophilic esophagitis in pediatrics: advances in diagnosis, treatment, and follow-up

**DOI:** 10.1016/j.jped.2026.101635

**Published:** 2026-10-29

**Authors:** Cristina Targa Ferreira, Elisa de Carvalho, Carlos M. Timossi, Luciana Rodrigues Silva, Marise Helena Cardoso Tofoli, Maria do Carmo Barros de Melo, Jackeline de Motta Franco, Lucila Camargo Lopes de Oliveira, Renata Rodrigues Cocco, Herberto José Chong-Neto, Dirceu Solé

**Affiliations:** a Brazilian Society of Pediatrics (SBP), Brazil; b Miramar MedCom, Department of Research and Development, Mexico City, Mexico; c Latin American Society of Pediatric Gastroenterology, Hepatology and Nutrition, Mexico City, Mexico; d Brazilian Association of Allergy and Immunology (ASBAI), Brazil; e Instituto Pensi, São Paulo, SP, Brazil

**Keywords:** Eosinophilic esophagitis, Proton pump inhibitor, Esophageal biopsy, Child, Dupilumab

## Abstract

**Objective:**

To identify the evidence and recommendations regarding pediatric eosinophilic esophagitis (EoE), including its diagnosis, treatment, and follow-up.

**Sources:**

Narrative review based on a comprehensive and structured search of the MEDLINE (PubMed), EMBASE (Ovid), LILACS, and SciELO databases using the terms “diagnosis” OR “clinical manifestations” OR “treatment” OR “follow-up” AND “eosinophilic esophagitis” AND “children” through March 2026. Data obtained from the Brazilian Consensus (Brazilian Society of Pediatrics/Brazilian Association of Allergy and Immunology) were incorporated.

**Summary of the findings:**

EoE is a chronic and emerging disease, and a significant cause of esophageal dysfunction. Early and accurate diagnosis is fundamental to avoid complications, such as fibrostenotic sequelae. Treatment can be performed with proton pump inhibitors, topical corticosteroids, and empirical diets, chosen according to clinical profile, adherence, and socioeconomic context. In refractory cases, dupilumab is an effective option. Allergy tests should not guide elimination diets. Endoscopic follow-up with biopsies is essential for evaluating response and maintenance, as the relapse rate is high, even in asymptomatic patients. Reassessment intervals should be individualized, but post-induction control between 8 and 12 weeks and periodic reviews during maintenance are recommended.

**Conclusions:**

The dissociation between symptoms and histological inflammation represents one of the greatest clinical challenges, in addition to the heterogeneity of therapeutic response and limitations of long-term studies. Changes in follow-up and treatment algorithms are expected, especially with the inclusion of dupilumab. The Brazilian consensus represents an attempt to standardize diagnostic criteria, approach, and treatment.

## Introduction

Eosinophilic esophagitis (EoE) is a chronic, immune-mediated inflammatory disease characterized by symptoms of esophageal dysfunction and the presence of eosinophilic infiltrate in the esophageal mucosa (15 or more eosinophils per high-power field - hpf), after exclusion of other causes of esophageal eosinophilia [[1], [2], [3]].

EoE is an emerging disease, and its prevalence has been increasing significantly worldwide [[1], [2], [3]]. Current estimates report incidences of 20 cases of EoE per 100,000 people, similar to that of inflammatory bowel disease, and that it is increasing mainly in adults [2]. Since its description in recent decades, a progressive increase in incidence and prevalence has been observed, possibly related to the interaction between environmental, genetic, and epigenetic factors [[1], [2], [3]].

In the pediatric context, EoE is a significant cause of esophageal dysfunction and gastrointestinal symptoms, such as difficulties in swallowing, chest pain, and reflux, making early and accurate diagnosis crucial to avoid long-term complications like esophageal stricture and impact on quality of life [[1], [2], [3]].

The pathophysiology of EoE is predominantly related to a type 2 immune response, not mediated by IgE, and involves cytokines such as interleukin (IL)-4 and IL-13. EoE can evolve from an inflammatory to a fibrostenotic phenotype, with a significant impact on quality of life. It can present with dysphagia, food impaction, and symptoms consistent with gastroesophageal reflux disease (GERD) [[1], [2], [3]].

The therapeutic approach to EoE involves three main pillars: pharmacological treatment, dietary therapy, and endoscopic interventions, with the more recent incorporation of biological therapies [[1], [2], [3], [4]]. Systematic follow-up of patients plays a central role in preventing fibrostenotic complications and detecting subclinical inflammatory activity, since symptoms do not reliably correlate with histological activity [[1], [2], [3], [4]].

In recent years, there have been significant advances in understanding the pathophysiology, natural history, diagnostic criteria, and therapeutic strategies of the disease. Important conceptual and therapeutic changes — such as the incorporation of proton pump inhibitor (PPI)-responsive eosinophilia within the EoE spectrum and treatment with biologics — have modified diagnostic algorithms and clinical practices [[1], [2], [3], [4]].

This article aims to identify current evidence and recommendations regarding pediatric EoE, covering its diagnosis, treatment, and follow-up.

## Materials and methods

This narrative review was based on a comprehensive and structured search of the medical literature on EoE. The search was conducted in the following databases: MEDLINE (PubMed), EMBASE (Ovid), LILACS, and SciELO. The search strategy incorporated a validated filter to identify randomized controlled trials, systematic reviews, and clinical practice guidelines, and included the following MeSH terms and keywords: “diagnosis” [MeSH Terms] OR “clinical manifestations” [All Fields] OR “treatment” [MeSH Terms] OR “follow-up” [All Fields] AND “eosinophilic esophagitis” [All Fields] OR “children” [All Fields] until March 2026. Studies involving pediatric populations that addressed diagnostic methods and therapeutic interventions were included. Articles in Portuguese, English, and Spanish were eligible. Case reports, studies limited to adult populations, and articles without direct relevance to the disease in its early stages were excluded. The study design, methodological quality, limitations, and clinical applicability were critically evaluated, and areas of consensus, controversy, and unmet research needs were integrated into the narrative synthesis. Data obtained from the Brazilian Consensus on EoE, conducted using the Delphi method, which included 20 pediatric gastroenterologists and 20 pediatric allergists affiliated with the Brazilian Society of Pediatrics and the Brazilian Association of Allergy and Immunology, were also incorporated [5,6].

### Definition, clinical picture and pathophysiology

EoE is unanimously defined as a chronic immune-mediated esophageal disease associated with symptoms of esophageal dysfunction [[1], [2], [3],^6^]. Histologically, it is characterized by 15 or more eosinophils/high-power field (eos/hpf) or 60 or more eos/mm² in at least six biopsies from the upper and lower levels of the esophagus, after excluding other causes of esophageal eosinophilia [[1], [2], [3],^6^].

The first reports of EoE occurred in the 1990s, when studies conducted with adult patients showed symptoms such as dysphagia associated with esophageal eosinophilia, demonstrating that it was not a rare condition [7,8]. In addition, Kelly et al. [9] published data from some patients with esophagitis and eosinophils in the esophagus who only responded to an elemental diet with an amino acid formula.

Initially, response to a PPI trial was thought to differentiate patients with EoE from those with GERDa, and only patients unresponsive to PPI therapy (or, alternatively, those with normal esophageal pH monitoring) could be diagnosed withwith EoE [4,10]. From 2017 and 2018 onward, PPI-responsive esophageal eosinophilia (PPI-REE) became synonymous withwith EoE [10,11]. It was found that patients with eosinophils in the esophageal mucosa, whether or not they responded to PPIs, had the same clinical, endoscopic, histological, molecular, and transcriptomic characteristics as patients with EoE; therefore, the diagnostic algorithm remained the same for all patients [4,10,11] (Figure 1).

**Fig. 1:**
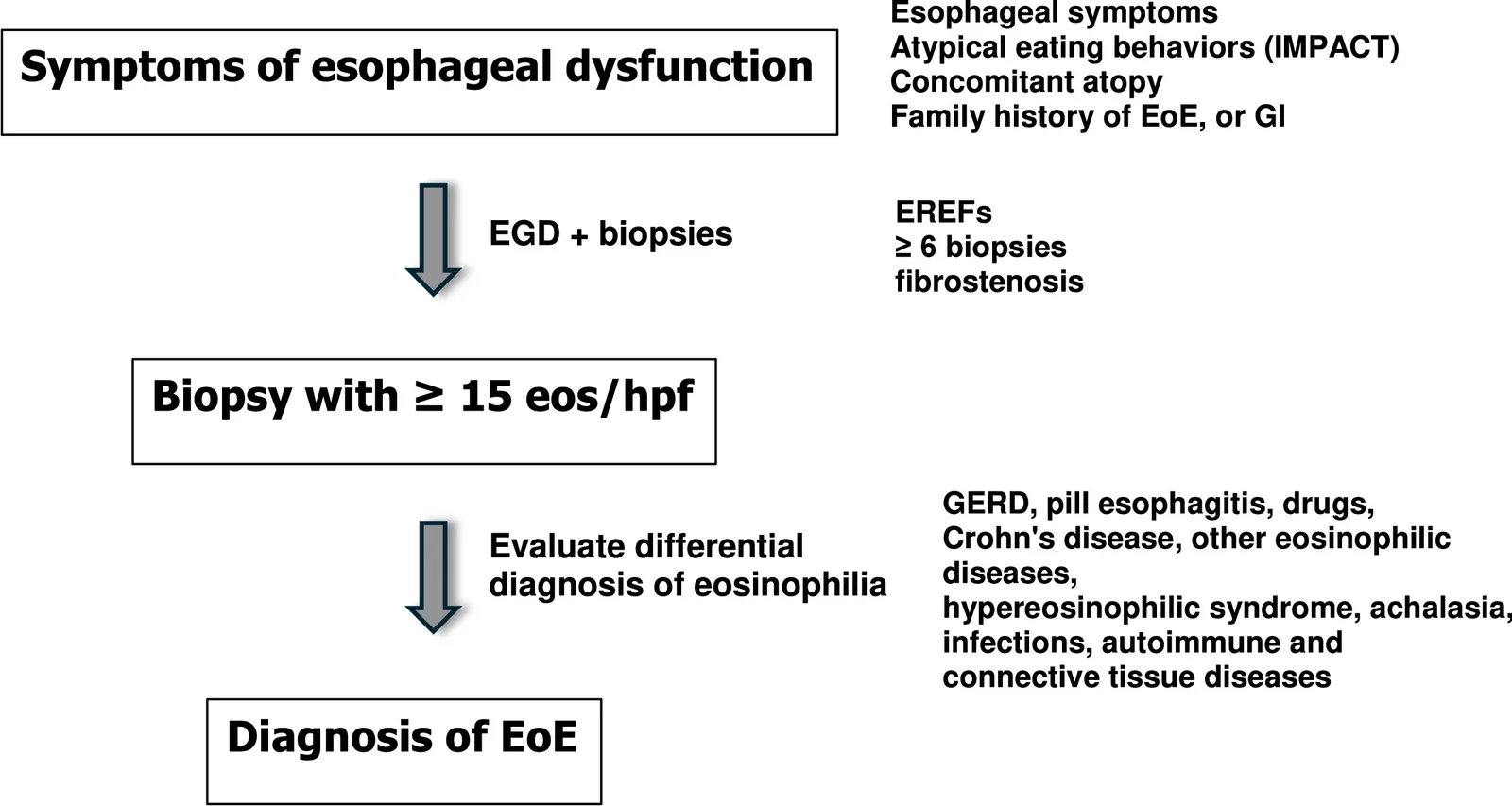
Diagnostic algorithm for eosinophilic esophagitis (EoE) (modified from Dellon et al. [3]). GI, gastrointestinal; EGD, esophagogastroduodenoscopy; EREFs, Eosinophilic Esophagitis Endoscopic Reference Score*;* eos/hpf, eosinophils/high power field; GERD, gastroesophageal reflux disease.

### Clinical picture

EoE can affect any age group, although symptoms may differ in children, adolescents, and adults [[1], [2], [3]]. EoE causes inflammation and dysmotility, which determine the symptoms. Infants and younger children generally present with more nonspecific symptoms similar to GERD; on the other hand, older children and adolescents often manifest dysphagia and food impaction, although heartburn and chest pain may also be present [[1], [2], [3],^12^]. In infants and young children, failure to thrive, difficulty progressing to solid foods, feeding refusal, abdominal pain, vomiting, regurgitation, heartburn, and acid reflux are common symptoms [12].

EoE causes well-known eating disorders. Both children and adults with EoE may exhibit feeding difficulties, such as delaying and consuming excessive liquids at mealtimes, eating only small pieces, chewing slowly, and using adaptive behaviors during meals [1,3]. In children, in particular, these adaptive behaviors include difficulties introducing new foods, food refusal, preference for liquids and soft, pureed foods, and increased meal times. Clinicians may use the acronym IMPACT to assess this type of eating behavior [12,13] (Table 1).

**Table 1:** Eating behaviors in patients with eosinophilic esophagitis. (IMPACT).

**I**mbibe fluids with meals
**M**odify food (cutting into small pieces, pureeing)
**P**rolong meal times
**A**void hard texture foods
**C**hew excessively
**T**urn away tablets, pills

Adapted from Hirano & Furuta [13].

The IMPACT tool is neither a diagnostic criterion nor an instrument for determining diagnostic probability based on the number of positive items. Rather, it is a clinical tool designed to identify compensatory behaviors that may mask dysphagia. The presence of one or more IMPACT behaviors should heighten clinical awareness of possible esophageal dysfunction in the appropriate clinical context; however, the tool itself is not a diagnostic criterion for EoE [12,13].

For standardized assessment of symptom severity in children with established or suspected EoE, the validated Pediatric Eosinophilic Esophagitis Symptom Score version 2.0 (PEESS v2.0) may be used, particularly at diagnosis and during assessment of treatment response and disease monitoring [1].

### Pathophysiology

EoE is a chronic immune-mediated disease triggered predominantly by food antigens in genetically susceptible individuals. Its pathophysiology results from the interplay among genetic factors, epithelial barrier dysfunction, environmental exposures, and a predominantly type 2 inflammatory response. Activation of the esophageal epithelium promotes the production of mediators that favor T helper type 2 (Th2) cell responses, with interleukin (IL)-4, IL-5, and IL-13 playing central roles [14]. Food antigens are the major recognized triggers of EoE. In addition to food antigens, aeroallergens have been implicated as potential modulators of EoE activity. The mechanisms underlying the loss or failure of immune tolerance to food antigens remain incompletely understood. The immune response appears to be predominantly T-cell mediated and is not dependent on IgE, although many patients have concomitant atopic diseases. Inflammatory T cells displaying a type 2 phenotype and repertoires associated with food antigens have been identified in patients with EoE, further supporting the antigen-specific nature of the disease. Epithelial barrier dysfunction may, in turn, increase antigen exposure and perpetuate this adaptive immune response, suggesting a bidirectional interaction between epithelial abnormalities and loss of immune homeostasis [15].

Persistent inflammation promotes progressive structural changes in the esophagus. Eosinophils, mast cells, and mediators such as transforming growth factor beta-1 (TGF-β1) contribute to fibroblast activation, extracellular matrix deposition, and tissue remodeling. In children, subepithelial fibrosis and increased TGF-β1 expression can be demonstrated early in the course of the disease, supporting the concept that uncontrolled inflammation may, over time, lead to reduced esophageal compliance and the development of rings, narrowing, and strictures [14].

Finally, factors associated with rapid environmental and dietary changes have been proposed to explain, at least in part, the increasing incidence of EoE. Changes in food processing, food additives, and greater consumption of ultra-processed foods have been proposed as potential modifiers of epithelial barrier function, the microbiota, and immune responses. However, direct evidence demonstrating a causal relationship between ultra-processed food consumption and EoE is currently insufficient. This hypothesis should therefore be regarded as an emerging area of investigation rather than an established etiologic mechanism [15].

### Treatment

The therapeutic goals in pediatric EoE include achieving clinical-histological and endoscopic remission, correction of feeding dysfunction and nutritional deficiencies, prevention of complications related to remodeling, and maintenance of an adequate quality of life, preserving the development and growth of these children [[12], [13], [14], [15], [16], [17]]. The treatment strategy needs to be agreed upon individually with the patient and caregivers, using a shared decision-making model, aiming to ensure a simpler, lower-cost, feasible approach adapted to each patient, with monotherapy recommended for treatment initiation (rather than combination therapy) and treatment effectiveness assessed after 8 to 12 weeks by endoscopy [17].

Established anti-inflammatory treatments for EoE include dietary therapy, PPIs, and swallowed topical corticosteroids, which may be combined with endoscopic dilation in patients with esophageal stenosis [16]. In this context, the ESPGHAN EGID WG suggests that a short course of systemic steroids be considered as an alternative to dilatation in the presence of moderate to severe esophageal strictures with severe symptoms [1].

Eosinophilic Diseases Working Group of ESPGHAN (*European Society for Pediatric Gastroenterology, Hepatology and Nutrition*) [1] recommends PPIs, empirical elimination diets, and topical steroids as first-line induction treatment options due to their efficacy and safety, with 100% expert approval and no evidence of preference for any of the three options. Other consensus statements and guidelines also support this position [[2], [3], [4],^12^] (Figure 2).

**Fig. 2:**
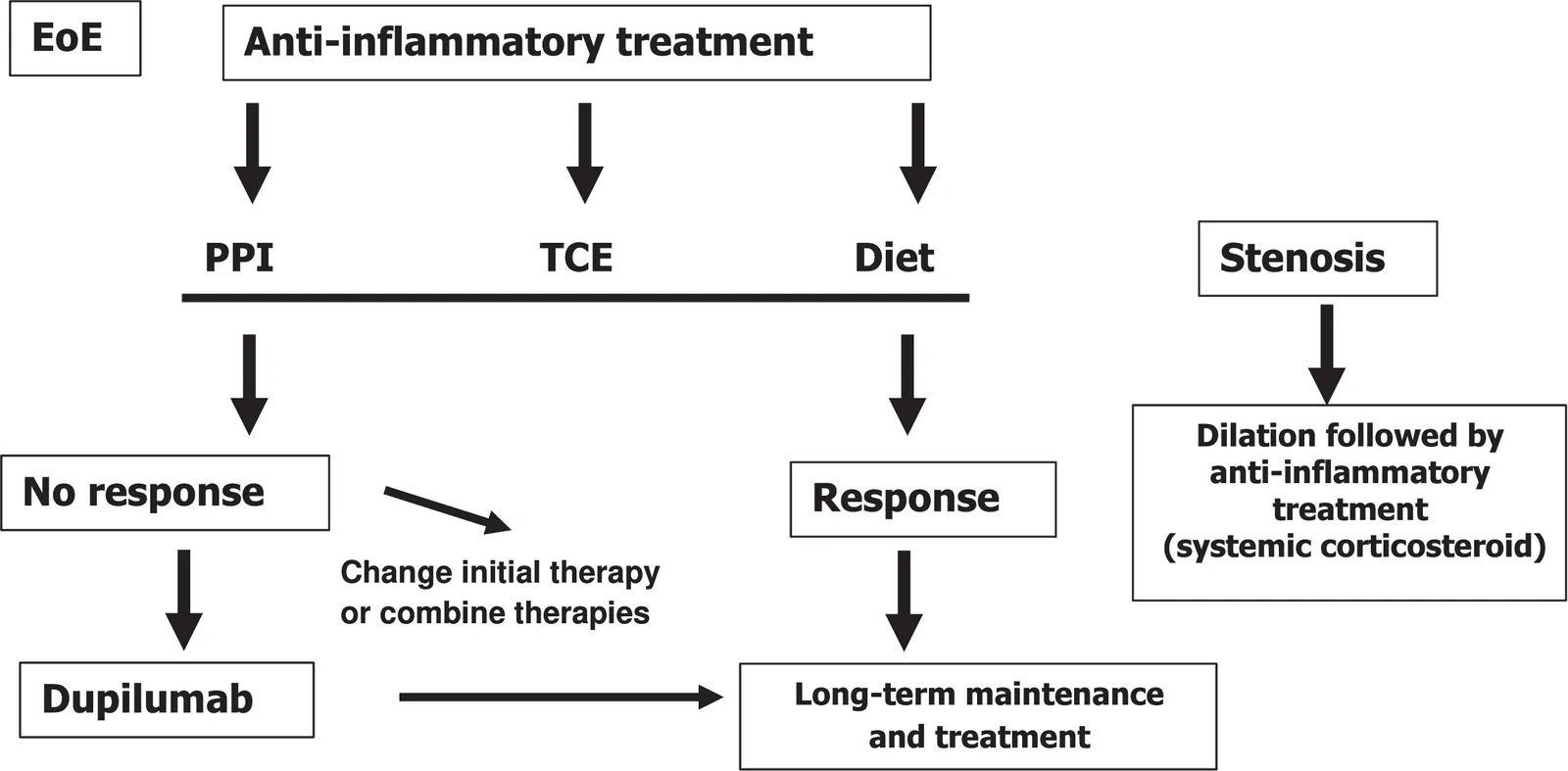
Suggested treatment algorithm for eosinophilic esophagitis (EoE). Modified from Dellon et al. [3]. PPIs: proton pump inhibitors; TEC: topical corticosteroids. *Note:* Increasing the dose, ruling out nonadherence, switching to another treatment, and combination therapy should be considered before switching to dupilumab in EoE in pediatric patients.

According to current consensus, without effective treatment, inflammation and symptoms tend to persist for a long time and may worsen, as the disease can lead to esophageal remodeling and esophageal stenosis [[1], [2], [3], [4]]. Even if remission is achieved after an initial course of treatment, relapse is almost inevitable within a variable timeframe once treatment is discontinued. This justifies not only induction therapy but also maintenance therapy, as recommended by the ESPGHAN EGID WG, which advises maintenance treatment for all patients after achieving histological remission [1]. The progressive nature of the disease has a significant impact on patients' health-related quality of life, clearly indicating the need for treatment, especially in the pediatric age group, when patients are still growing and developing [3].

According to the American College of Gastroenterology, growth (height and weight), development (including feeding skills), and nutrition (adequate nutrient intake) are among the symptoms of EoE in children. As such, they should be included as treatment goals, alongside the goals of symptomatic, endoscopic, and histological improvement [3].

The increasing prevalence of EoE has generated great interest in research into new therapeutic approaches. Investigations into new topical corticosteroid formulations targeting the esophagus and modern therapeutic approaches aimed at blocking the molecular pathways that cause inflammation in EoE have been published [16]. New therapeutic approaches include monoclonal antibodies (dupilumab, mepolizumab, reslizumab, benralizumab, and cendakimab), as well as JAK-STAT (Janus kinase blockers – JAK, signal transducer and activator of transcription proteins - STAT), and S1PR agonists (Sphingosine-1-phosphatereceptors), among others [16]. Some have demonstrated short-term efficacy and safety, but without maintenance of these effects. Some biologics seems definitely withdrawn concerning EoE as they are eosinophil-depleting biologics, leading to nearly complete esophageal depletion in EoE but without symptom improvement and with persistent signs of disease activity (i.e. omalizumab- anti IgE mAb, mepolizumab-anti IL5 mAb, lirentelimab- anti Siglec-8 mAb, benralizumab- anti IL-5ra mAb, reslizumab- anti IL5 mAb) [[1], [2], [3],^16^]. It is always important to discuss the treatment options available in the studied country for the pediatric population [6].

As efficacy is quite similar to first-line therapy, the choice of treatment should initially be agreed upon with the patient and may change over time. A change in therapy may be necessary in the long term due to treatment side effects, lack of patient adherence to a diet or medication, or when there is a negative impact on the patient's quality of life or family resources. Healthcare professionals should consider the quality of life of both the patient and the family when selecting a treatment plan for children with EoE. These considerations will likely impact the feasibility as well as adherence to the prescribed treatment [4,18].

In developing countries, patients may face financial constraints and even difficulties understanding treatment. Diets are often difficult in the pediatric age group and impractical when implemented for long periods, compromising adherence [6,18]. Prescribing medication twice a day also hinders long-term treatment, and corticosteroids can influence growth, especially in patients with multiple comorbidities who receive various types and administrations of corticosteroids [1,3,6,18]. All of this must be considered when establishing therapeutic goals, in addition to the characteristics of the disease in each patient [3,6,16,17].

### Dietary treatment

Allergy test-guided elimination diets have, in the past, been a theoretically attractive alternative to empirical elimination and elemental formulas. However, EoE is a delayed-type 2 lymphocyte-mediated hypersensitivity, in which IgE is dispensable, and consequently, studies with elimination diets based on specific IgE research (skin tests or quantification of serum levels) have had limited success in predicting food triggers of EoE [3]. Considering the lack of precision of available allergy tests, particularly skin prick tests, patch tests, and quantification of serum IgE or IgG in predicting food triggers in EoE, and with lower response rates compared to empirical elimination diets, allergy test-based diets are not currently recommended, and serum IgE panels for foods should not be requested in the evaluation of EoE [[1], [2], [3]]. Therefore, empirical dietary elimination remains the only viable approach for patients willing to try dietary therapy [17]. It is worth reminding that endoscopy is necessary to confirm remission and the efficacy of the treatment, regardless of the treatment used. Therefore, endoscopy is mandatory after each dietary restriction adopted and after each food rechallenge.

Empirical food elimination diets can induce clinical and histological remission in a significant proportion of patients with EoE when guided by a qualified professional (as depicted in Table 2), although long-term adherence may be challenging for patients [1,2,3,6,18,19].

**Table 2:** Efficacy of histological remission according to different types of elimination diet.

Type of Diet	Foods Eliminated	Success (Historical)
**Elemental Diet**	Amino Acid Formula	> 90%
**6 foods**	Milk, Wheat, Egg, Soy, Nuts and Peanuts, and Fish	40% - 70%
**4 foods**	Milk, Wheat, Egg and Soy	40% - 50%
**2 foods**	Milk and dairy products and Wheat	40% - 45%
**1 food**	Only cow's milk and dairy products	35% - 45%

Adapted from Dellon et al. [3].

Over the past decade, there has been a gradual transition from highly restrictive diets to less restrictive initial diets, which is very promising and increases patient satisfaction [1].

Recently, Kliewer et al. [20] demonstrated that, although the four-food elimination regimen moderately improved symptoms in patients with EoE compared to the one-food (cow's milk) regimen, the histological, endoscopic, quality of life, and transcriptomic results were similar in both groups. They state that a one-food restriction diet is a reasonable first-line therapy option for pediatric EoE, considering its effects, tolerability, and relative simplicity [20].

Studies suggested that a single-food (cow's milk) elimination diet is a reasonable initial treatment option, which reduces endoscopies and improves patient adherence [6,17,19] (Table 2).

### Proton pump inhibitors

Treatment with PPIs, as monotherapy, has demonstrated efficacy in a varying proportion of patients with EoE in different studies [1,3,4,18,21]. PPIs have the potential to be an effective primary therapeutic option due to their long history of safety and ease of administration. Patients may choose to start PPI treatment before considering corticosteroids or exclusion diets [22]. The different responses observed by different groups may reflect not only differences in study designs, but also variations in severity, administered doses, or genetic, phenotypic, and/or environmental factors [18].

Gutiérrez-Junquera et al. [23], in their first prospective pediatric study, observed nearly 80% clinical improvement and 69% histological improvement with twice-daily PPI treatment. This emphasizes that symptom resolution may not reflect the objective (histological) response to PPI therapy and that a second (follow-up) endoscopy with esophageal biopsies is necessary to diagnose clinical-histological response [17,22]. These same authors demonstrated, in a pediatric population, up to 70% clinical and histological remission after one year of follow-up with half the initial PPI dose (1 mg/kg/day) [24,25].

In accordance with recent studies, double doses of PPIs in patients from Western countries were significantly superior to standard or single doses, while quadruple doses (e.g., omeprazole 40 mg twice daily) did not result in additional clinical or histological benefit [20,26].

A study conducted in Southern Brazil with patients with EoE aged between one month and 18 years who received PPIs as first-line treatment found that 70% responded to PPIs, with 22% showing a complete response and 48% showing a partial response [18] (Table 3) [[27], [28], [29], [30], [31], [32], [33], [34], [35]].

**Table 3:** Clinical and histological efficacy of proton pump inhibitors (PPIs) in pediatric studies.

Author, year	Design	N	IBP	Dose	Duration (without)	Histological Response histological (%)	70% Clinical response (%)
Sayej et al., 2009 [27]	R	36	L, E, O	2mg/kg/day	12	39	78
Dranove et al., 2009 [28]	R	43	NR	NR	NR	40	86
Schoeder et al., 2010 [29]	R	35	NR	1–2 mg/kg/day	8	23	23
Rea et al., 2013 [30]	P	25	NR	NR	8	60	NR
Lucendo et al., 2017 [31]	RS	619/188^&^	Variable	Variables/study	Variable	50.5	61
Gutierrez-Junquera et al., 2016 [23]	P	51	E	2mg/kg/day - BID	8	69	82
Gómez-Torrijos et al., 2016 [32]	P	34	O	2mg/kg/day - BID	8	26.5	NR
Harris et al., 2018 [33]	R	64	NR	High doses	NR	41	NR
Vieira et al., 2020 [34]	R	231	E, O, Pa	2mg/kg/day - BID	8	28	NR
Rosen et al., 2021 [35]	R	94	NR	2mg/kg/day - BID	NR	44	55
Gutierrez-Junquera, 2018[25]	P	387	E, L, O	1–2 mg/kg/day - BID	8 - 12	51	88
Nader et al., 2023[18]	P	27	E, O	2mg/kg/day - BID	8 - 12	70	96

R, retrospective; L, lansoprazole; E, esoprazole; O, omeprazole; NR, not reported; P, prospective; RS, systematic review; &, children; Pa, pantoprazole; BID, twice daily.

PPIs were more effective in prospective studies than in retrospective studies (53%vs. 39%) and when administered twice daily compared to once daily (56%vs. 50%), but without statistical significance [3]. Based on available evidence, the recommended doses of PPIs to induce remission of EoE in children are 1 to 2 mg/kg of omeprazole daily or equivalent [1,3,4,18,20].

Lucendo et al. [21], in 2025, conducted a systematic review and meta-analysis of 73 studies, comprising 7304 patients treated with PPIs for remission induction and maintenance in EoE. PPI therapy resulted in clinical response in 65% and histological remission in 45% of patients, with no differences between children and adults [21]. Overall, 34% achieved <5 eos/hpf. Histological remission was significantly greater with double doses of PPIs compared to the standard dose. The response was significantly greater in studies with a lower risk of bias. Half the maintenance dose resulted in sustained histological remission in 68% of patients [21].

A Spanish multicenter study (RENESE *registry*), which constitutes the largest pediatric series to evaluate the efficacy of PPIs, documented evidence of PPI treatment efficacy in this large cohort of pediatric patients with EoE, who achieved sustained histological remission with a dose-reduction strategy [36]. Patients who achieved complete initial histological remission were more likely to achieve long-term histological remission [36]. In the same study, a favorable safety profile of PPI therapy was observed, with adverse events reported in only 2.4% of children. All adverse events were mild and transient, and treatment interruption was necessary in only two patients. The safety of PPI therapy for children is still somewhat controversial [36]. They conclude that PPI therapy induces clinical-histological remission in almost half of pediatric and adult patients with EoE [19]. The response to PPIs is variable, depending on the site studied. For all these reasons, PPIs become a more inclusive option, especially in developing areas, as they are less expensive and more feasible, being a treatment applicable to this population [6,17].

EUREOS study [37], one of the largest practical studies defining the effectiveness of EoE treatment in real-world pediatrics, showed that PPI monotherapy was the preferred first-line treatment option in pediatric patients with EoE, with overall efficacy similar to dietary options but inferior to steroid therapy. Treatment choice depended on patient age (with diets being more commonly used in younger children), endoscopic phenotypes at the start of treatment (PPIs being preferred in patients with predominantly inflammatory features on initial endoscopy), and the patient's country of origin (with PPIs being predominantly prescribed as a first-line option in Spain) [37].

PPIs have the potential to be an effective primary therapeutic option due to their long history of safety and ease of administration. Patients may choose to start PPI treatment before considering corticosteroids or exclusion diets [20]. The recommended doses of PPIs to induce remission of EoE in children are 1 to 2 mg/kg of omeprazole daily or equivalent [1,3,4,18,21].

### Swallowed topical corticosteroids

Topical corticosteroids (viscous budesonide or swallowed fluticasone) are effective in inducing clinical and histological remission, with multiple randomized clinical trials demonstrating benefit [1,3,4,6]. They can also be considered first-line treatment [1,3,4,6]. Evidence points to: superior histological efficacy compared to PPIs and diets in real-world studies; histological response of approximately 60% to 65%; and a favorable safety profile [1,3,4,6]. Steroids were preferentially used by some authors in patients with a mixed/stenosing phenotype and when fibrotic features were found on baseline endoscopy [37].

In the prospective, real-world cohort, EuropEEr [38], 173 of 583 pediatric patients with EoE received topical oral corticosteroid monotherapy after failure to respond to PPIs. Of these, 71% achieved symptomatic remission and 59% histological remission with this treatment [1,38]. Also in the CONNECT-EUREOS study, clinical and histological response rates to PPIs, corticosteroids, and diets used in monotherapy were evaluated and compared (Table 4) [37]. There were no differences in overall clinical response, being 72%, 86%, and 73%, respectively [37]. Regarding histological outcomes, steroids were the most effective, with 65% of patients achieving < 15 lt; 15 eos/hpf, higher than the 43% and 42% with PPIs and diets, respectively [37]. Finally, and in agreement with these results, the clinical-histological response (clinical improvement along with < 15 lt; 15 eos/hpf) was better for topical steroids (66%) than for PPIs and diets (44% and 42%, respectively), with most patients (60%, 35%, and 28%) achieving clinical-histological remission (improvement of symptoms and < 5 lt; 5 eos/hpf in esophageal biopsies) [37].

**Table 4:** First-line therapies and histological and clinical response rates for each treatment.

Clinical and histological response rates	IBP (%)	Steroids swallowed (%)	Exclusion diets (%)	Total (%)	P
**Clinical improvement**	72	86	73	74	0.178
**Histological remission (< 5 lt; 5 eos/hpf)**	34	60	29	37	0.017
**Histological response (< 15 lt; 15 eos/hpf)**	9	4	13	9	
**Clinical-histological remission**	35	60	28	37	0.001
**Clinical-histological response**	44	66	42	47	0.018

Adapted from Navarro et al. [37].

Specific formulations of topical corticosteroids for EoE exist in the United States and Europe; in Brazil, compounded or adapted preparations are used (fluticasone delivered via a metered-dose nasal, swallowed, rather than inhaled, and budesonide compounded as a viscous oral suspension (slurry/gel) mixed with a sweetener).

They are also recommended for maintenance, although the ideal dose and duration are not yet defined. Relapse is frequent after discontinuation for all three treatments [6]. In children, attention should be paid to steroid overdose, represented by the sum of doses administered for asthma and/or atopic dermatitis and/or EoE (inhaled, nasal, cutaneous, systemic) that may be associated [6].

Regarding adverse effects, the following stand out: oral/esophageal candidiasis (2% to 15%) and possible adrenal suppression (rare, but monitorable, especially in patients undergoing multiple treatments) [6]. The EoETALY Consensus suggests careful clinical monitoring of possible treatment-related adverse events in patients using topical steroids long-term [4].

The use of swallowed steroids requires special recommendations: attention must be paid to the proper methods of administering topical corticosteroids orally, ensuring they are ingested at least 10 min after eating or drinking, and maintaining a fast for 30 to 60 min after ingestion [1,4]. Topical corticosteroids delivered via metered-dose inhaler should be administered directly into the mouth and not via a spacer, as recommended for asthma (nor via Diskus or Turbohaler, as administration of the medication requires inhalation). Topical steroid suspensions, such as viscous budesonide, should be administered slowly orally to allow the medication to settle in the esophagus and not enter the stomach as a single bolus [1,4]. The recommended dosee of budesonide is 0.5 to 2 mg twice daily and the recommended dose of fluticasone spray is 88 to 500 µg twice daily.

Eosinophilic Diseases Working Group recommends that the total steroid load be calculated and considered (e.g., systemic, topical, nasal, or inhaled) when treating EoE with topical steroids, as the combination may lead to increased cumulative steroid exposure and possible adrenal insufficiency, although rare [1].

### Combined treatment

The combination of two or more therapies is not recommended as a routine initial strategy [6,37]. Combination therapies may be considered in therapeutic failures and after initial trial with monotherapies [6,37].

### Systemic corticosteroids

Systemic corticosteroids are indicated for short periods in cases of moderate or severe stenotic disease, especially when immediate access to dilation is limited. Pediatric studies show a high resolution rate for inflammatory stenosis with short courses of treatment [1]. The shorter duration of disease in most children, compared with adults, seems to suggest that inflammatory stenosis, rather than fibrostenotic stenosis, is more common and, therefore, that severe pediatric stenoses may respond well to short courses of systemic corticosteroids [1]. In this setting, immediate endoscopy is often not possible; therefore, the use of systemic steroids can improve patient symptoms [1]. Most studies have shown that mild inflammatory stenoses associated with pediatric EoE resolve with standard drug treatment (PPIs and/or corticosteroids) or even with dietary treatment.

A retrospective study of 20 children with moderate to severe EoE and strictures that prevented the passage of a standard or pediatric endoscope showed that a short course of systemic corticosteroids (two to four weeks, followed by rapid reduction to standard treatment) resulted in symptomatic improvement in all patients and complete endoscopic resolution of the stricture in 95% [39]. Only mild side effects were reported: hyperphagia (10/20), weight gain (5/20), hyperactivity (2/20), and acne (1/20). Mechanical esophageal dilation was required in 3/20 patients during a median follow-up of 48.5 months [1,39].

### Esophageal dilation

It is safe and effective for the treatment of fibrotic sequelae and persistent dysphagia [[1], [2], [3], [4]]. Esophageal perforation rates are very low. Esophageal dilation should always be associated with anti-inflammatory therapy, since dilation acts on the underlying inflammation.

According to ESPGHAN, endoscopic evaluation and barium contrast esophageal examination are complementary to the evaluation of suspected strictures or narrow esophagus in patients with EoE [1]. Some data have been published in children with EoE, in whom 55% of the strictures observed on barium esophagogram were not detected endoscopically [40].

### Treatment with immunobiological agent – dupilumab

Dupilumab is the only biological treatment to date that has scientific evidence of improvement in children with EoE [6]. It is a fully humanized anti-IL-4 α-receptor antibody that inhibits IL-4 and IL-13 signaling and has demonstrated benefits in cases of moderate to severe atopic dermatitis, asthma, and chronic sinusitis with nasal polyposis [1,3,4,6].

In Brazil, dupilumab was approved by ANVISA in April 2023 for individuals aged 12 and older (weighing ≥ 40 kg) and, in November 2024, for children aged one year and older weighing 15 kg or more [41].

A recent randomized, double-blind, phase 3 clinical trial in adolescents over 12 years of age and adults included two arms: dupilumab 300 mg weekly versus placebo and dupilumab 300 mg every two weeks versus placebo. Children/adolescents over 12 years of age and weighing 40 kg participated in this study [42]. In conclusion, the study indicated that in patients with EoE, weekly subcutaneous administration of dupilumab improved histological findings and alleviated disease symptoms.

Next, another study in children older than one year and weighing >15 kg was published [43]. Based on the positive results of this phase 3 study in children aged 1 to 11 years (EoE KIDS), the Food and Drug Administration (FDA) recently approved dupilumab treatment for this age group as well [43]. In this age group, the recommended dose is 200 or 300 mg every two weeks.

ESPGHAN recommends that dupilumab can be used in selected cases of children older than one year of age, weighing >15 kg, with EoE refractory to conventional treatment, and in those with concomitant atopic burden, with approved indications for the biologic [1]. The other biologic medications tested and under study have not yet been proven or approved for pediatrics [1,3,4,12].

A retrospective, real-world cohort study in adults with EoE refractory to standard therapies, most with severe esophageal stenosis and requiring multiple prior esophageal dilatations, found histological response rates after dupilumab of 80% and 57%, respectively, for <15 eos/hpf and ≤ 6 eos/hpf [44]. There was also a significant increase in esophageal diameter before dilation, accompanied by symptom improvement [44].

Due to its high cost and the still frequent difficulty in obtaining it in developing countries, dupilumab is recommended primarily for more severe cases or those unresponsive to other treatments, or when EoE is associated with other atopic conditions, such as asthma and significant atopic dermatitis. Patients who are non-compliant or do not follow other treatments may also be candidates for dupilumab (Table 5) [1].

**Table 5:** Medication options for the treatment of eosinophilic esophagitis [1].

Drug	Age group	Induction dose: non-stricturing and mild symptoms^1^	Induction dose: stricturing or severe symptoms^1^	Initial maintenance dose (usually half of the induction dose for 3–6 months)^a^^,1^
Budesonide^b^^,^^c^	Infant 1–3 years	0.5 mg × 1/d–0.5 mg × 2/d	0.5 mg × 2/d	0.25 mg × 1/d–0.5 mg × 2/d
	Child 4–10 years	1 mg × 1/d	1 mg × 2/d	0.5 mg × 1/d–1 mg × 1/d
	Adolescent 11–18 years	1 mg × 2/d	1 mg × 2/d–2 mg × 2/d	0.5 mg × 1/d–1 mg × 1/d
Fluticasone propionate ¶^,^^b^^.^^c^	Infant 1–3 years	88 µg × 1/d–250 µg × 1/d	88 µg × 2/d–250 µg × 2/d	44 µg × 1/d–125 µg × 1/d (in some cases 125 µg × 2/d)
	Child 4–10 years	250 µg × 1/d–250 µg × 2/d	500 µg × 1/d–500 µg × 2/d	250 µg × 1/d–500 µg × 1/d
	Adolescent 11–18 years	500 µg × 1/d–500 µg × 2/d	500 µg × 2/d–500 µg × 3/d	500 µg × 1/d
PPIs (given as omeprazole dose—adjust to equivalent dosing of any PPI)	Infant 1–3 years	1 mg/kg × 2/d	1 mg/kg–2 mg/kg × 2/d^d^	1 mg/kg × 1/d
	Child 4–10 years	1 mg/kg × 2/d (maximum 60 mg/d)	1 mg/kg–2 mg/kg × 2/d (maximum 60 mg/d) d	0.5 mg–1 mg/kg/d (maximum 30 mg)
	Adolescent 11–18 years	1 mg/kg × 2/d (maximum 80 mg/d)	1 mg/kg–2 mg/kg × 2/d (maximum 80 mg/d) d	1 mg/kg × 1/d (morning dosing) (maximum 40 mg/d)
Dupilumab	> 1 year and 15–30 kg	200 mg every 2 weeks	200 mg every 2 weeks	200 mg every 2 weeks
	30–40 kg	300 mg every 2 weeks	300 mg every 2 weeks	300 mg every 2 weeks
	> 40 kg	300 mg weekly	300 mg weekly	300 mg weekly

Other options: Esomeprazole (20 and 40 mg); Pathoprazole (20 and 40 mg); Lansoprazole (15 and 30 mg); Dexlansoprazole (30 and 60 mg).

§ - Formulated with honey or sucralose and thickener (0.5 mg, 1 mg, 2 mg).

Note: From “Diagnosis and management of eosinophilic esophagitis in children: An update from the European Society for Paediatric Gastroenterology, Hepatology and Nutrition (ESPGHAN)," by Amil-Dias et al.^1^, 2024, Journal of Pediatric Gastroenterology and Nutrition, vol. 79, issue 2, p. 394–437. Copyright 2026 by John Wiley & Sons, Inc. Adapted with permission (RightsLink Copyright License No 6360941060379).

PPI, proton-pump inhibitor.

¶ - Flixotide® (50,125,250 mg/ puff); Flixonase® (50mcg/dose).

a After 3–6 months, another dose titration may be considered on an individual basis (often another decrease of dose by 25–50%).

b For once-daily dosing, nighttime dosing is recommended to extend contact time of the drug with the esophageal wall. The drug should be taken after tooth brushing, and optimally no food or drink should be given for 1 h. If twice-daily dosing, give the morning dose after breakfast.

c Fluticasone multidose inhalers are supplied at different doses in different countries; therefore, dosing may vary slightly depending on availability.

d PPIs seem to be inferior to topical steroids at inducing remission in the presence of strictures and therefore are not recommended. If signs of concomitant reflux esophagitis are present, a low-dose PPI together with a topical steroid may be considered initially.

### Patient follow-up and monitoring

International and Brazilian consensus guidelines establish complete agreement that patients with EoE should be monitored regularly, considering three main aspects: symptoms, endoscopic findings, and histological activity [[1], [2], [3], [4]]. The natural history of the disease demonstrates a high rate of relapse after treatment interruption, regardless of the therapeutic modality used [[1], [2], [3], [4]]. Even patients in clinical and histological remission should maintain periodic follow-up, as fibrotic progression can occur in the absence of evident symptoms [[1], [2], [3], [4]].

Upper digestive endoscopy with esophageal biopsies is considered the gold standard for monitoring EoE activity [[1], [2], [3], [4]], since consensus guidelines emphasize that isolated symptoms do not adequately reflect histological inflammation.

Endoscopy with biopsies should be performed 8 to 12 weeks after beginning treatment; repeat endoscopy after each therapeutic change, and early endoscopic reassessment in case of symptomatic recurrence, are consensus recommendations [2,6,45]. There is limited data on specific follow-up intervals for reassessment of patients with EoE on maintenance therapy [4].

There is strong evidence that treatment interruption is associated with increased inflammatory activity, greater endoscopic severity, reduced esophageal caliber, and a higher risk of stenosis [46].

A retrospective study conducted with 701 adult patients with EoE showed that a gap in care exceeding two years was associated with increased disease activity and fibrostenotic complications, especially in those who did not receive regular follow-up [46]. Compared with the pre-treatment interruption period, patients in the post-interruption period presented greater endoscopic severity and smaller esophageal diameters [46]. Stenosis was more prevalent with longer intervals between assessments [46]. Each additional year of interval increased the odds of stenosis by 26%, even after considering pre-treatment interruption dilation [46]. Furthermore, of the 67 patients without pre-interruption fibrosis, 25 (37%) presented at least one fibrotic feature (stenosis, narrowing, or need for dilation) in the post-interruption period [46].

Based on the available evidence, it is proposed that patients with EoE who are stable on maintenance treatment or in clinical remission, or with histological remission confirmed in their last upper endoscopy, undergo a clinical evaluation 12 to 18 months after the last endoscopy to assess the status of symptoms and possible side effects related to treatment [1,4,46]. In addition, repeat endoscopy with esophageal biopsies to assess the histological activity of the disease should be discussed on a case-by-case basis, when clinically indicated and based on the risk of asymptomatic recurrence of the disease [4].

EoE treatment may be required at any point, and the current trend is not to interrupt it [6].

Dose reduction may be considered in patients in remission [1]. ESPGHAN suggests endoscopic and histological reassessment after one to three years, during the maintenance phase, in cases of stable clinical remission [1]. ESPGHAN also suggests that decisions to maintain, reduce, or discontinue treatment should be determined by the severity of the disease and the specific needs of the patient [1]. It is acknowledged that knowledge about this condition is still being built, and it is possible that new guidelines will emerge in the future [1].

## Conclusions

EoE is a chronic and emerging disease affecting the esophagus, characterized by eosinophilic inflammation, and its prevalence has increased significantly worldwide [[1], [2], [3], [4]]. EoE has changed from a rare disease to a condition frequently encountered by gastroenterologists and allergists in emergency rooms and endoscopy centers, often because of food impactions. However, questions remain regarding whether its prevalence will continue to increase [47].

In the pediatric context, EoE is a significant cause of esophageal dysfunction, with symptoms affecting swallowing and feeding, as well as gastrointestinal symptoms such as difficulty swallowing, chest pain, and reflux. Early and accurate diagnosis is crucial to avoid long-term complications such as esophageal stricture and impact on quality of life [[1], [2], [3], [4]]. PPIs, topical corticosteroids, and empirical diets represent first-line therapies, chosen according to clinical profile, adherence, and socioeconomic context [1,4]. Dupilumab emerges as an effective option for refractory cases and patients with multiple atopic diseases [1,4,3,42,43]. Allergy tests should no longer guide elimination diets [1,3,4]. Esophageal dilation is safe for fibrostenotic sequelae [1,3,4,37]. Endoscopic follow-up with biopsies remains essential for evaluating response and maintenance [1,3,4].

The high relapse rate after treatment discontinuation and the risk of fibrostenotic progression support the recommendation for maintenance therapy and regular follow-up, even in asymptomatic patients [1,3,4,48,49]. Reassessment intervals should be individualized, but post-induction monitoring between 8 and 12 weeks and periodic reviews during maintenance are strongly recommended. Evidence of fibrostenotic progression associated with lack of continuous follow-up modifies the traditional approach based solely on clinical response [48,49]. The current model prioritizes sustained inflammatory control and objective surveillance.

The standardization of diagnostic criteria - especially the incorporation of PPI response within the EoE spectrum - reduces historical ambiguities and improves clinical consistency [10]. The requirement for clinicopathological correlation reinforces the need for an integrated approach between gastroenterology and allergology [1,3,4].

The SBP–ASBAI consensus [6] represents an attempt to standardize the approach to pediatric EoE and establishes clear diagnostic criteria and a structured methodology for treatment. A relevant point is the adaptation of the recommendations to the Brazilian reality, considering resource availability, costs, and variability in care scenarios. The shared decision-making model is emphasized as an essential component of care.

The recommendations reinforce that EoE should be treated individually as a structurally progressive chronic inflammatory disease [1,3,4,50]. The dissociation between symptoms and histological inflammation represents one of the greatest clinical challenges, justifying the systematic use of endoscopy with biopsies in follow-up [1,3,4,[48], [49], [50]]. Another relevant point is the lack of consensus on ideal maintenance regimens, reflecting heterogeneity of therapeutic response and limitations of long-term studies. The recent incorporation of biological therapies, such as dupilumab, suggests that changes in follow-up and treatment algorithms are expected in the coming years [[42], [43], [44]].

The general pediatrician plays an important role in the early recognition and long-term care of children and adolescents with EoE. Awareness of suggestive manifestations—including feeding difficulties, food refusal, persistent vomiting, dysphagia, food impaction, and impaired growth — may facilitate earlier diagnosis and reduce diagnostic delay. Pediatricians should monitor nutritional status, growth, feeding behavior, and treatment adherence, while maintaining close collaboration with pediatric gastroenterologists and other members of the multidisciplinary team. Referral to a pediatric gastroenterologist is recommended whenever EoE is suspected, particularly in patients with persistent or recurrent dysphagia, food impaction, significant feeding difficulties, growth or nutritional impairment, symptoms refractory to conventional treatment, or other clinical features suggesting esophageal dysfunction. Referral to a pediatric allergist is important when another type 2 disease is associated, such as asthma or atopic dermatitis. Specialist evaluation is essential for diagnostic confirmation through upper gastrointestinal endoscopy with esophageal biopsies, selection and adjustment of therapy, assessment of histological response, and management of persistent or complicated disease.

## Funding

This research did not receive any specific grant from funding agencies in the public, commercial, or not-for-profit sectors.

## Declaration of generative AI and AI-assisted technologies in the manuscript preparation process

The authors did not use generative AI or AI-assisted technologies in the preparation of this manuscript.

## Institution for indexing (Index/ MEDLINE)

Brazilian Society of Pediatrics (SBP), Brazil.

## Authors’ contributions

All authors made substantial contributions to the manuscript as follows:

Conception and design of the review: Cristina Targa Ferreira, Elisa de Carvalho, Luciana Rodrigues Silva, Dirceu Solé.

Literature search and data acquisition: Cristina Targa Ferreira, Elisa de Carvalho, Carlos M. Timossi, Marise Helena Cardoso Tofoli, Maria do Carmo Barros de Melo, Jackeline de Motta Franco, Lucila Camargo Lopes de Oliveira, Renata Rodrigues Cocco, Herberto José Chong-Neto.

Drafting the article: Cristina Targa Ferreira, Elisa de Carvalho, Dirceu Solé.

Critical revision for important intellectual content: all authors.

Final approval of the version to be submitted: all authors.

## Conflicts of interest

CHTF has worked as a consultant for Sanofi, Danone, Nestlé, Aché, and Apsen. MHCT has worked as a consultant for Ache, Apsen, Cifarma, Danone, Ipsen, Nestlé, Sanofi. RRC and HJCN have worked as a consultant for Sanofi. The other authors have no conflicts of interest to declare.

Cristina Targa Ferreira: received speaker fees and/or research support from Sanofi, Danone, Nestlé, Aché, and Apsen.

Marise Helena Cardoso Tofoli: received speaker fees and/or research support from Aché, Apsen, Cifarma, Danone, Ipsen, Nestlé, and Sanofi.

Renata Rodrigues Cocco: received speaker fees and/or research support from Sanofi.

Herberto José Chong-Neto: received speaker fees and/or research support from Sanofi.

All other authors declare no conflicts of interest.

## Data Availability

This is a narrative review article. No primary dataset was generated. All data discussed are from previously published studies cited in the reference list.
